# Individual and community-level risk factors of women’s acceptance of intimate partner violence in Ethiopia: multilevel analysis of 2011 Ethiopian Demographic Health Survey

**DOI:** 10.1186/s12905-021-01427-w

**Published:** 2021-08-04

**Authors:** Emiru Merdassa Atomssa, Araya Abrha Medhanyie, Girmatsion Fisseha

**Affiliations:** 1 Department of Public Health, Institute of Health Sciences, Wollega University, Nekemte, Oromia Ethiopia; 2 School of Public Health, College of Health Sciences, Mekelle University, Mekelle, Tigray Ethiopia

**Keywords:** Acceptability of IPV, Individual-level effects, Community-level effects

## Abstract

**Background:**

The prevalence of Intimate partner violence (IPV) is higher in societies with higher acceptance of norms that support IPV. In Ethiopia, the proportion of women’s acceptance of IPV was 69%, posing a central challenge in preventing IPV. The main objective of this study was to assess the individual and community-level factors associated with women’s acceptance of IPV.

**Methods:**

Two-level mixed-effects logistic regression was applied to the 2011 Ethiopia Demographic and Health Survey data. A total of 16,366 women nested in the 596 clusters were included in the analysis.

**Results:**

The acceptability of the IPV was estimated to be 69%. Among the individual-level factors: women’s education with secondary and above (AOR = 0.38; 95% CI 0.29–0.52), partner’s education secondary and above (AOR = 0.71; 95% CI 0.54–0.82), women aged 35–49 years (AOR = 0.67; 95% CI 0.54–0.82), fully empowered in household level decision making (AOR = 0.67; 95% CI0.54–0.81), literate (AOR = 0.76; 95% CI 0.62–0.92), and perceived existence of law that prevents IPV (AOR = 0.56; 95% CI 0.50–0.63) were significantly associated with women’s acceptance of IPV. Similarly, rural residence (AOR = 1.93; 95% CI 1.53–2.43) and living in the State region (AOR = 2.37; 95% CI 1.81–3.10) were significantly associated with the women’s acceptance of IPV among the community-level factors.

**Conclusion:**

Both individual and community-level factors were significant risk factors for the acceptability of intimate partner violence. Women's education, women's age, women’s empowerment, partner education level, perceived existence of the law, and literacy were among individual factors. State region and residence were among community-level risk factors significantly associated women’s acceptance of IPV.

## Background

Intimate partner violence (IPV) is any behavior within an intimate relationship that causes physical, psychological, or sexual harm to a current or former partner or spouse since the age of 15 [1, 2]. Overall, 30% of women worldwide and 45.6% of women in Africa experience lifetime prevalence of IPV [1]. In 2005, the World Health Organization conducted the study in ten selected countries: Bangladesh, Brazil, Ethiopia, Japan, Namibia, Peru, Samoa, Serbia and Montenegro, Thailand, and the United Republic of Tanzania [3]. This study reported the highest prevalence of IPV in Ethiopia with a lifetime prevalence (71%) and 12-months prevalence (54%). Similarly, the previous studies conducted in different parts of Ethiopia showed that the lifetime and past 12 months prevalence of IPV were also high [4–6].

IPV impacts are wide-ranging, resulting in immediate and long-term adverse health outcomes [7, 8]. It affects educational and economic under-performance, unsafe sexual practices, reduced ability to bond as part of parenthood, and increased uptake of health-risk behaviors such as alcohol and illicit drug use [9]. Not only does IPV devastate the lives of women, but it also incurs great costs to the society as a whole. The global economic costs of IPV, including healthcare costs, were estimated to be 4423 billion USD per year, which is approximately 5.18% of Gross Domestic Product (GDP) [10]. The GDP lost due to IPV-related absenteeism was estimated at 1.5% of the overall economy, including male and female lost days [11].

Several studies have shown that IPV is always rooted in social, cultural, and attitudes about what is acceptable or what is not acceptable in an intimate relationship [12–14] and some factors increase and create an acceptable climate for violence [3]. The fundamental change to the social attitudes are vital to respond effectively to this problem and reducing the acceptability of all forms of IPV against women has become one of the fundamental goals of public health [15].

In 1993, the United Nations general assembly adopted a landmark declaration on the elimination of violence against women [16]. The acceptability of IPV has been identified as the main reason for delaying the elimination of violence against women (VAW) [17, 18]. IPV against women is not only a major social and public health problem but also largely undereported: causing an inability to estimate the real magnitude of the problem [19, 20].

The prevalence of IPV is higher in societies that have higher women's acceptance of IPV [21]. In Ethiopia, the prevalence of IPV acceptance was 68% posing a central challenge in preventing IPV [22]. This acceptance contributed to the social climate in which IPV against women is tolerated and legitimized. This lifelong pattern of justifying abusive behaviors and immature self-concepts predisposes women to victims by their partners who seek to fill their power and control needs through disempowerment [22]. This makes IPV eradication difficult.

Acceptance of IPV is a complex problem that needs to be understood within the broader social context, including the family and community [23, 24]. Previous studies have also recommended that research in this area is limited and needs to be conducted by considering the hierarchical nature of the problem [15, 25]. Hence, further research is required to explore factors associated with Women’s acceptance of IPV using a multilevel approach. Apart from these, existing literature was limited, inconsistent findings, and not representative of the whole population [25–34]. Thus, this study aimed to answer the following questions: Are individual and community-level factors associated with women’s acceptance of IPV? Do communities differ in women’s acceptance of Intimate partner violence? Do factors explain the community-level variance in women’s acceptance of IPV?

## Methods

### Study setting and data source

Ethiopia is the study area. Administratively, Ethiopia is divided into nine regional states: Tigray, Afar, Amhara, Oromia, Somali, Benishangul, SNNPR, Gambella, Harari and two city administrations: Addis Ababa and Diredawa. The data source is the nationally representative 2011 Ethiopia Demographic Health Survey (EDHS). The survey was a population-based cross-sectional study designed to provide population and health indicator estimates at national and regional levels, as well as urban and rural residents.

### Sample size and sampling procedures

Data from the EDHS 2011 were used, specifically data on individual women of childbearing age. All eligible women in the 624 clusters were the study population. The sample was selected using a stratified, two-stage cluster design and enumeration areas (EAs) were the sampling units for the initial stage of sampling. The sampling frame was a list of all EAs established from the population and housing census in 2007. The first stage involved the selection of clusters. The second stage involved the selection of households from the selected clusters. Following the above procedures at the first stage, the sample contained 624 EAs, but 28 of the clusters were not interviewed because of the drought and security problems in the Somali region. In the second stage, a representative sample of 17,817 households was selected for the survey with 17,385 eligible women identified for individual interviews, and 16,515 women were interviewed. To gain interpretability of results, those who answered don't know and had a missing response for all justifications were excluded. These exclusions resulting in a loss of only 149 (0.9%) women and giving a final sample of 16,366 for the analysis.

### Study variables and measurements

In lower-income countries, including Ethiopia women’s acceptance of IPV were measured using attitudes toward IPV scale of measurement as recommended by the DHS measure [35]. The justification was measured in each survey question by assessing response (yes/no) to five attitudinal scenarios/questions. Women were asked if they felt a husband would be justified in beating his wife if she: goes out without telling him, neglects the children, argues with him, refuses to have sex with him and burns the food. Responses to these questions were transformed into a single dichotomous "Yes" or "No" variable. Women who responded "Yes" to one or several of the questions formed and were coded as **Yes** (1) and women who responded "no" to all the questions coded as **No** (0).

The independent variables were socio-economic and demographic characteristics of the respondents (women’s education, literacy, partner education, education difference, partner's occupation, women’s occupation, owning a house, wealth index, ever chewed chat, alcohol consumption, women’s autonomy, marital status, family system, women’s age, age at first sex, age at first cohabitation, partner age, number of living children, cohabitation duration, pregnancy status; cultural factors: ethnicity and religion); psychosocial factors (perceived existence of law); and Community-level factors (community literacy, community poverty, community media, community residence and State region).

Women empowerment is measured by women’s participation in household decision making concerning who decides on: women's health care, large household purchases, visits to family or relatives and how men's earnings are used were measured in the DHS. If the woman decided jointly with her partner or by herself, she was assigned as participated in decision making and did not otherwise. Further, a new variable 'women empowerment' was created by assuming participation as a proxy measure of women empowerment and leveled into: Empowered if she is involved in four of the decision making, Partially empowered if involved in one of the decisions, two of the decision and three of the decision, and not empowered if not involved in any decision.

Community-level variables were created by aggregating individual's characteristics within their clusters. They were computed using the proportion of selected levels of a given variable that were concerned with per cluster. Since the aggregate values for all generated variables have no meaning at the individual-level, they were categorized into groups based on the national median values. Through this aggregation, the proportion of community factors ranging between 0 and 50th percentiles were categorized as low, and the range between 50 and 100th percentiles were categorized as high. Median values were used because of the non-normality of aggregated variables. Community poverty was constructed from the first two lower quintiles (poorest and poor) as proportions, and distinguishing clusters with low (0–50th percentiles) and high level of community poverty (50–100th percentiles). This procedure was also applied to create community-level factors for community media exposure considering the proportions of community members who have been exposed to any media (listening to the radio, watching television, reading magazines or newspapers) and community literacy (proportion of individuals who were able to read the whole sentence among women in the specified cluster). The two non-aggregate community-level factors included: residence (urban and rural), and contextual region dichotomized into city administration and State region.

### Statistical analysis

The DHS variable recode was designed to standardize variables that would make cross-country analysis easier and comparable. Distribution and values for each variable were assessed to detect implausible values and missing data values managed accordingly. Data were cleaned and analyzed using STATA software version 12.0. Data were examined and summarized using frequency and percent and presented using a table and bar graph. To get a reliable estimate data was given weight to adjust for differences in the probability of selection and non-response. Bivariate multilevel mixed-effects binary logistic regression was used for analyzing the association between explanatory variables and women’s acceptance of IPV. Variables with a p-value less than 0.05 in the bivariate analyzes were candidates for the multivariate analysis.

Multivariate two-level mixed-effects logistic regression was applied to the data to predict a binary outcome variable from a set of individual and community-level independent variables. The 2011 EDHS data present a clear multilevel structure and multilevel modeling used to permit the inclusion of error terms that reflect the variation pattern introduced by the data's hierarchical structure. Therefore, this analytic method was employed to account for the hierarchical structure of the data, in which 16,366 individuals (level 1) nested within 596 community groups (level 2).

The proportions of total variance related to community level factors were estimated by the intraclass correlation coefficient (ICC). The proportional change in variance (PCV) is the percentage reduction from the estimated variance in the null model as a result of included independent variables in the model. Results of fixed effects were interpreted with an adjusted odds ratio (AOR) with a 95% confidence interval (95%CI). The random effect was interpreted using ICC and PCV and compared across the progressive models by looking at them.

The interaction effect was checked and there was no interaction effect (“Appendix 3”). Moreover, the multicollinearity was also checked by using variance inflation factors (VIF) and no variable had VIF > 10 [36, 37]. Akaike information criterion (AIC) was used to compare models with different sets of parameters. A model with the lowest Akaike Information Criteria (AIC) was considered as the best fit model.

### Data quality assurance

Standard model questionnaires were designed and developed by the DHS program with the basic approach of collecting quality data. Developed English version questionnaires were translated into three major languages Amharigna, Afan Oromo, and Tigrigna. Complete interviews were conducted, yielding a response rate of 95%.

## Results

### General background characteristics of study respondents

**Fig. 1:**
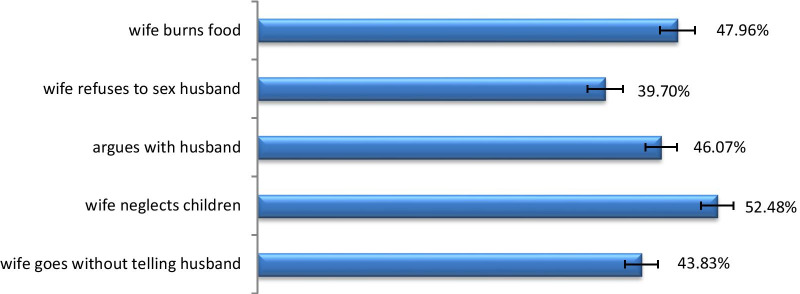
Reason for accepting IPV among women 15–49 years Ethiopia DHS, 2011

In the study sample, 69% of the women were accepted IPV. Almost half of the women 8303 (50.8%) had no education and nearly half of the women 5018 (49.7%) were fully empowered in household decision making. There were 596 clusters which the number of women in each cluster ranged from 5 to 59. Fifty five percent (n = 317) of the clusters had a higher poverty status (Table1). The most frequent reason reported for the women’s acceptance of IPV was (52.50%). when women neglected children The least frequent reason reported was (39.70%) when women refused to have sex with their husbands (Fig. 1).

**Table 1:** Characteristics and percentage distribution of women of childbearing age 15–49, accepting attitude of IPV by selected characteristics using 2011 EDHS, Ethiopia

Variables	Frequency (%)	
	Unweighted	Weighted
*Women's education*		
No education	8201 (50.11)	8303 (50.83)
Primary	5807 (35.48)	6211 (38.02)
Secondary and above	2358 (14.41)	1820 (11.15)
*Women's age*		
15–24	6778 (41.42)	6846 (41.91)
25–34	5246 (32.05)	5156 (31.57)
35–49	4342 (26.53)	4332 (26.52)
*Religion*		
Orthodox	6929 (42.71)	7745 (47.82)
Muslim	6107 (37.64)	4542 (28.04)
Others	3189 (19.65)	3910 (24.14)
*Currently pregnant*		
No	15,095 (92.23)	15,138 (92.67)
Yes	1271 (7.77)	1196 (7.33)
*Age at first sex*		
No sex before	3896 (23.83)	4085 (25.04)
< 15	3175 (19.42)	3515 (21.54)
15–17	4936 (30.19)	4702 (28.82)
18 and above	4343 (26.56)	4012 (24.60)
*Women empowerment*		
Underpowered	1028 (10.33)	883 (8.76)
Partially empowered	4131 (41.52)	4187 (41.50)
Fully empowered	4791 (48.15)	5018 (49.74)
*Women has occupation*		
No	7912 (48.80)	6919 (42.67)
Yes	8301 (51.20)	9296 (57.33)
*Number of living children*		
No child	5686 (34.74)	5607 (34.32)
1–3	5931 (36.24)	5702 (34.92)
4–6	3572 (21.83)	3646 (22.32)
7 and above	1177 (7.19)	1379 (8.44)
*Partner education level*		
No education	5856 (49.45)	5901 (49.94)
Primary	4072 (34.39)	4560 (38.59)
Secondary and above	1914 (16.16)	1355 (11.47)
*Partner age*		
15–24	612 (6.06)	648 (6.37)
25–34	3339 (33.09)	3339 (32.82)
35–49	4259 (42.21)	4268 (41.95)
50 and above	1881 (18.64)	1919 (18.86)
*Education difference*		
The same	5621 (47.49)	5661 (47.91)
Less than him	4674 (39.49)	4629 (39.19)
Greater than him	1541 (13.02)	1524 (12.90)
*House owning*		
No	7398 (45.23)	6931 (42.47)
Yes	8957 (54.77)	9389 (57.53)
*Wealth index*		
Poor	6063 (37.05)	5970 (36.55)
Middle	2251 (13.75)	3009 (18.42)
Rich	8052 (49.20)	7355 (45.03)
*Family system*		
Monogamous	8777 (87.07)	9080 (89.48)
Polygamous	1303 (12.93)	1068 (10.52)
*Perceived existence of law against IPV*		
No	8632 (52.77)	8315 (50.92)
Yes	7727 (47.23)	8014 (49.08)
*Literacy*		
Illiterate	11,491 (70.41)	11,747 (72.19)
Literate	4829 (29.59)	4526 (27.81)
*Community mass media exposure*		
Low	312 (52.35)	268 (46.59)
High	284 (47.65)	308 (53.41)
*Community residence*		
Urban	184 (30.87)	135 (23.47)
Rural	412 (69.13)	441 (76.53)
*Community region*		
City administration	96 (16.11)	31 (5.32)
State region	500 ( 83.89)	546 (94.68)
*Community poverty*		
Low	286 (47.99)	259 (44.99)
High	310 (52.01)	317 (55.01)
*Community literacy*		
Low	313 (52.52)	297 (51.60)
High	283 (47.48)	279 (48.40)
*IPV justified*		
No	5662 (34.60)	5032 (30.81)
Yes	10,704 (65.40)	11,302 (69.19)

### Bivariate analysis

The highest percentage of the acceptance of IPV was reported in women who had no education (78.91%) compared to the women who had a secondary or higher education level (34.20%). Similarly, the acceptance of IPV varies according to the husband's education level. The highest percentage of women's acceptance of IPV was observed among those whose husbands' had no education (85.51%). Women’s acceptance of IPV varies according to their wealth index. The proportion of acceptance of IPV was (80.21%) among women who were poor compared to women who were rich (57.24%). The proportion of women’s acceptance of IPV was higher (71.90%) in the State region compared to the city administration. The proportion of acceptance of IPV was higher among women who were living in rural areas (76.25%). Hence, the acceptance of IPV varies by clusters where women were living. Women who live in the low literacy cluster had higher (79.10%) acceptance of IPV than women who live in high literacy clusters (Table 2).

**Table 2:** Characteristics and percentage distribution of women of childbearing age 15–49, accepting attitude of IPV by selected characteristics using 2011 EDHS, Ethiopia

Variables	IPV accepted		Crude OR (95% CI)
	No	Yes	
*Women education*			
No education	20.19	79.81	1
Primary	34.74	65.26	0.56 (0.52–0.62)
Secondary and above	65.80	34.20	0.20 (0.17–0.23)
*Women age*			
15–24	34.35	65.65	1
25–34	29.58	70.42	1.10 (1.01–1.20)
35–49	26.67	73.33	1.20 (1.09–1.32)
*Religion*			
Orthodox	35.08	64.92	1
Muslim	28.90	71.10	1.68 (1.48–1.92)
Others	24.83	75.17	1.27 (1.09–1.49)
*Currently pregnant*			
No	31.52	68.48	1
Yes	21.85	78.15	1.17 (1.01–1.36)
*Age at first sex*			
No sex before	40.85	59.15	1
< 15	21.29	78.71	1.73 (1.53–1.95)
15–17	25.95	74.05	1.52 (1.37–1.69)
18 and above	34.41	65.59	1.14 (1.03–1.27)
*Women empowerment*			
Underpowered	11.76	88.24	1
Partially empowered	19.90	80.10	0.90 (0.73–1.10)
Fully empowered	33.00	67.00	0.49 (0.40–0.61)
*Women has occupation*			
No	30.56	69.44	1
Yes	31.01	68.99	0.94 (0.87–1.02)
*Number of living children*			
No child	39.59	60.41	1
1–3	30.21	69.79	1.24 (1.13–1.35)
4–6	21.81	78.19	1.66 (1.49–1.85)
7 and above	21.36	78.64	1.54 (1.30–1.82)
*Partner education level*			
No education	19.49	80.51	1
Primary	26.71	73.29	0.71 (0.64–0.79)
Secondary and above	56.77	43.23	0.31 (0.27–0.37)
*Partner age*			
15–24	20.72	79.28	1
25–34	25.49	74.51	0.84 (0.66–1.06) *
35–49	27.39	72.61	0.81 (0.65–1.02)
50 and above	24.80	75.20	0.85 (0.66–1.08)
*Education difference b/n wife & Husband*			
The same	21.52	78.48	1
Less than him	29.82	70.18	0.81 (0.73–0.90)
Greater than him	35.19	64.81	0.65 (0.57–0.76)
*House owning*			
No	42.11	57.89	1
Yes	22.51	77.49	1.58 (1.45–1.72)
*Wealth index*			
Poor	19.79	80.21	1
Middle	23.45	76.55	0.90 (0.78–1.02)
Rich	42.76	57.24	0.51 (0.45–0.58)
*Family system*			
Monogamous	26.23	73.77	1
Polygamous	21.98	78.02	1.07 (0.91–1.27) *
*Perceived existence of law against IPV*			
No	21.61	78.39	1
Yes	40.37	59.63	0.50 (0.46–0.54)
*Literacy*			
Illiterate	22.43	77.57	1
Literate	52.08	47.92	0.36 (0.33–0.39)
*Community mass media exposure*			
Low	20.93	79.07	1
High	38.87	61.13	0.26 (0.22–0.32)
*Community residence*			
Urban	53.42	46.58	1
Rural	23.75	76.25	5.69 (4.76–6.80)
*Community region*			
City adminstration	74.29	25.71	1
State region	28.10	71.90	6.39 (5.00–8.16)
*Community poverty*			
Low	41.39	58.61	1
High	21.68	78.32	3.71 (3.07–4.48)
*Community literacy*			
Low	20.90	79.10	1
High	40.84	59.16	0.24 (0.20–0.29)

### The multilevel multivariate logistic model

**Fig. 2:**
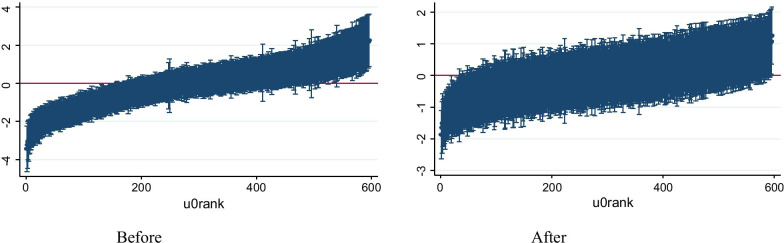
Caterpillar plot before and after predictor variables (individual-level and community-level) entry to the model

Four models were built, the first was the null, the second was individual-level variables, the third was community-level variables, and the fourth was the combined (models II and III) which were significant at *p* < 0.05. Table 3 presents the multilevel multivariate logistic regression analysis results in which individual characteristics and community-level factors were assessed. The first step in the multilevel modeling was to consider if the data justified the decision to assess random effects at the cluster level. We first fit a simple model (null model) with no covariates in the model, that is, an intercept-only model that predicts the probability of acceptance of IPV. There was a significant variation in the odds of accepting IPV across the clusters (ICC = 0.32, σ^2^u0 = 1.57, *p* < 0.001). This shows both individual and community-level variables are important in explaining women’s acceptance of IPV. The random intercept model variance decreased compared to the random effect of the intercept empty model, from 32% in the empty model to 12% in the combined model (model 4), which was attributed to the inclusion of women's and community-level variables (Table 3). The combined model showed that 70% of the variance in women's acceptance of IPV was explained by individual and community-level factors. The reduction of community-level variance was depicted in "caterpillar" plots for shrunken residuals (logarithmic odds ratios) after adjusting for both individual and community-level predictors (Fig. 2). Multicollinearity was checked using the variance inflation factor (VIF); all of the covariates had VIF value less than 10, confirming that there was no indication for severe multicollinearity (“Appendix 3”). The AIC values of progressive models were computed and compared. Among the candidate models, the final fitted model with the least value of AIC 9698.32 (Table 3).

**Table 3:** Multivariate two-level mixed-effects logistic regression of women aged 15–49 years, acceptability of IPV in 2011 EDHS, Ethiopia

Variables	Model-I	Model-II AOR (95% CI)	Model-III AOR (95% CI)	Model-IV AOR (95% CI)
*Women's education*				
No education		1		1
Primary		0.77 (0.64–0.92)		0.77 (0.66–0.89)
2^nd^ and above		0.35 (0.25–0.48)		0.38 (0.29–0.52)
*Women's age*				
15–24		1		1
25–34		0.69 (0.58–0.82)		0.74 (0.62–0.88)
35–49		0.62 (0.50–0.77)		0.67 (0.54–0.82)
*Religion*				
Orthodox		1		
Muslims		1.04 (0.89–1.23)		
Others		1.24 (1.02–1.51)		
*Currently pregnant*				
No		1		
Yes		1.00 (0.85–1.18)		
*Age at first sex*				
No sex before		1		
≤ 14		0.39 (0.06–2.42)		
15–17		0.36 (0.06–2.22)		
18+		0.33 (0.05–2.02)		
*Women empowerment*				
Underpowered		1		1
Partially empowered		1.06 (0.87–1.30)		1.07 (0.87–1.31)
Fully empowered		0.64 (0.52–0.78)		0.67 (0.54–0.81)
*Number of living children*				
no child		1		
1–3		0.95 (0.78–1.15)		
4–6		1.17 (0.93–1.48)		
7 and above		1.10 (0.83–1.46)		
*Partner education level*				
No education		1		1
Primary		0.71 (0.57–0.87)		0.86 (0.75–0.98)
2nd and above		0.53 (0.39–0.72)		0.71 (0.54–0.82)
*Education difference*				
Same as husband		1		1
Less than husband		1.28 (1.04–1.58)		1.26 (1.02–1.56)
Greater than husband		1.21 (0.98–1.49)		1.21 (0.98–1.50)
*House owning*				
No		1		1
Yes		1.50 (1.30–1.72)		1.00 (0.81–1.25)
*Wealth index*				
Poor		1		1
Middle		1.06 (0.89–1.25)		1.07 (0.91–1.27)
Rich		0.72 (0.62–0.84)		0.90 (0.76–1.06)
*Perceived existence of law against IPV*				
No		1		1
Yes		0.54 (0.48–0.60)		0.56 (0.50–0.62)
*Literacy status of women*				
Illiterate		1		1
Literate		0.69 (0.56–0.84)		0.76 (0.62–0.92)
*Community region*				
City adminstration			1	1
State region			2.67 (2.09–3.40)	2.37 (1.81–3.10)
*Community media exposure*				
Low			1	
High			0.86 (0.70–1.06)	
*Community residence*				
Urban			1	1
Rural			2.43 (1.94–3.06)	1.93 (1.53–2.43)
*Community poverty*				
Low			1	1
High			1.39 (1.04–1.86)	1.40 (1.05–1.87)
*Community literacy*				
Low			1	1
High			0.66 (0.54–0.80)	0.98 (0.79–1.22)
*Random effect measure*				
ICC	0.32	0.17	0.14	0.12
PCV	Reference	56.40	67.04	70.00
*Model fitness*				
AIC	18,239.75	9825.51	17,780.76	9698.32

### Measures of associations (fixed effects)

In this study, a multilevel multivariate binary logistic regression model was employed. The results of the fixed part of the random coefficient model showed that women's education, women's age, husband's education, women's empowerment, perceived existence of law against IPV, Literacy of women, community poverty, place of residence, and contextual region and residents were significantly associated with acceptance of IPV among community-level factors (Table 3).

Independent of other factors, compared to women no education, primary (Adjusted odds ratio [AOR] 0.77; 95% CI 0.66–0.89) and secondary and above (AOR = 0.38; 95% CI 0.29–0.52) were less likely to have accepted IPV. Further, the results showed that women's whose husbands had a primary education level (AOR = 0.86; 95% CI 0.75–0.98) and secondary and above (AOR = 0.71; 95% CI 0.54–0.82) had lower odds of justifying IPV compared to women whose husbands had no education. Compared with women aged 15–24 years, women aged 25–34 years (AOR = 0.74; 95% CI 0.62–0.88) and 35–49 years (AOR = 0.67; 95% CI 0.54–0.82) were less likely to have accepted IPV.

The odds of accepting IPV were less likely for women who were fully empowered than women who were unempowered in domestic decision making (AOR = 0.67; 95% CI 0.54–0.81). Women who thought or perceived the existence of a law that prevents IPV were less likely to have accepted IPV than women who didn’t (AOR = 0.56, 95% CI 0.50–0.63). Literate women were less likely to have accepted IPV (AOR = 0.76, 95% CI 0.62–0.92) when compared to illiterate women.

Compared with women from the city administration, women from the State region (AOR = 2.37, 95% CI 1.81–3.10) were more likely to have accepted IPV. Residence was also significantly associated with acceptance of IPV (AOR = 1.93, 95% CI 1.53–2.43). See Table 3.

## Discussion

The study set out to investigate individual-level and community-level risk factors of Women’s acceptance of IPV. Both individual-level and community-level factors are important predictors of women’s acceptance of IPV. The multilevel logistic regression analysis result showed that women's education, women's age, husband’s education, women empowerment, literacy, and perceived existence of law were the main predictors among individual-level predictors, and contextual region and residents were significantly associated with accepting attitude of IPV among community-level factors.

This study showed that women with higher education levels had lower odds of accepting IPV. Some of the previous studies were comparable to this finding [27, 33]. In contrast to a study conducted in four provinces of Philippines showed that women's education had no significant association with IPV acceptance. In that study, a small sample size was used which might have contributed to the difference [28]. Women’s husbands who had a higher education level had lower odds of IPV acceptance than women whose husbands had no education. This showed that education could help women understand what is right about IPV and strengthen their attitudes that support victim safety and personal relevance to make appropriate decisions.

In this study, the likelihood of women’s acceptance of IPV was less for older women. This finding is consistent with the results of different studies undertaken in Asian countries and Africa [25, 38]. Early life and socialization might influence them to accept IPV [39] and possibly young women closer to the family for witnessing parental violence, had higher odds of accepting IPV [40].

Women's empowerment was a protective factor against acceptance of IPV. Women who had fully empowered in domestic decision making were less likely to have accepted IPV compared to underpowered women. This finding was similar to those of studies conducted in the Niger Delta and Bangladesh [31, 34]. Empowerment might contribute to the increasing confidence to justify what is acceptable to them and might influence women's views toward equality in a relation, rather than accepting violence. Women's empowerment is vital, as is changing social norms and notions of masculinity associated with power and dominance.

In this study, the literate woman was shown to be less likely to have accepted IPV than illiterate women. The evidence from a comparative study conducted in two countries, Kenya and Zambia supported this finding [41]. This might be because literate women have better access to information and education, which might influence and shape women's attitudes and learn what is acceptable and unacceptable.

This study also investigated contextual factors of women's acceptance of IPV. Women living in rural areas were more likely to have accepted IPV than women residing in urban areas. This is similar to studies conducted in Sub-Saharan Africa [38, 42, 43]. The contextual region was also significantly associated with women’s acceptance of IPV. There were regional differences in the odds of acceptance of IPV. This might be because the State regions were more likely to have accepted IPV compared to City administrations. State regions were less urbanized, educated women, and had low media exposure compared to the city administration. In addition, dissimilarity might be due to the contribution of different factors specific to the region (community norms, beliefs, customs, and others) that may explain the differences.

Th study findings were interpreted within the context of some study limitations and strengths. This study might be influenced by self-reported measures of attitudes and unavailability of important variables in 2011 EDHS data, such as the history of childhood abuse, women's family history, beliefs, and other cultural factors [24]. This study utilized cross-sectional data as there is no evidence of a temporal relationship between risk factors and women’s acceptance of IPV. This study conducted nationally representative data, which enables the generalisability at national level. This study also provides important insights into both individual and contextual factors influencing accepting attitudes of IPV using appropriate statistical modeling.

## Conclusions

This study suggests that both individual and community-level risk factors substantially affect the acceptance of IPV in Ethiopia. Women's education, women's age, women’s empowerment, partner education level, perceived existence of the law, and literacy were among the individual factors. State region and residence were among community-level risk factors that significantly associated the acceptance of IPV.

## Appendixies

### Appendix 1

See Table 4.

**Table 4:** Interaction and confounding effect test output

Suspected variables	Level	β Coef without x	β Coef with x	Δβ coef	Percent (wot-wz)/wz*100%	*P *value	Result	
Product terms							Interaction	Confounding
Cohabdur*weduc	1	− 0.1798017	− 0.2219209	− 0.0421192	18.97937508	NS		
	2	0.1071532	0.0312903	− 0.0758629	− 242.4486183			
Litstat*weduc	1	− 0.6564755	− 0.2822647	0.3742108	− 132.5744239	NS		
Cohabdur*wage	1	− 0.3284976	− 0.2219209	0.1065767	− 48.024634	NS		
	2	− 0.2366632	0.0312903	0.2679535	856.3468551			
Litstat*wempoerement	1	− 0.3699463	− 0.2822647	0.0876816	− 31.06360802	NS		
Empower*perexilaw	1	0.0055333	0.0720158	0.0664825	92.3165472	NS		
	2	− 0.4729046	− 0.4063732	0.0665314	− 16.371995			
Cohabdur*perexilaw	1	− 0.235816	0.2354489	0.4712649	200.1559149	NS		
	2	0.0046744	− 0.2219209	− 0.2265953	102.1063361			
Weduc*litstat	1	− 0.3513471	− 0.2676568	0.0836903	− 31.26776529	NS		
	2	− 1.218405	− 0.9687526	0.2496524	− 25.77050116			
Meduc*resid	1	− 0.1899441	− 0.154473	0.0354711	− 22.96265367	NS		
	2	− 0.4400745	− 0.3446921	0.0953824	− 27.67176851			
Ownhouse*resid	1	0.30346	0.2354489	− 0.0680111	− 28.88571575	NS		

*NS* non-significant

### Appendix 2

See Table 5.

**Table 5:** Model estimation using maximum likelihood estimation using numeric integrations

Variables	Levels	Coet	Integration points (adaptive Gaussian quadrature)							
			1	2	3	4	5	6	7	15
	Intercept	β0	0.4099206	0.4107573	0.4101662	0.4080258	0.4081808	0.4081286	0.4081079	0.4081097
	change	Δ	0.44	0.65	0.50	− 0.02	0.02	0.00	0.00	0
Weduc	1	β1	− 0.2621837	− 0.2621892	− 0.2620715	− 0.2619532	− 0.2619635	− 0.2619598	− 0.2619586	− 0.2619587
		Δ	0.09	0.09	0.04	0.00	0.00	0.00	0.00	0
	2	β2	− 0.9546576	− 0.954226	− 0.9532883	− 0.9532141	− 0.9532083	− 0.9532001	− 0.9532014	− 0.953201
		Δ	0.15	0.11	0.01	0.00	0.00	0.00	0.00	0
Meduc	1	β3	− 0.1525055	− 0.1525159	− 0.1522229	− 0.151932	− 0.1519509	− 0.1519412	− 0.151938	− 0.1519382
		Δ	0.37	0.38	0.19	0.00	0.01	0.00	0.00	0
	2	β4	− 0.3358535	− 0.3357814	− 0.335662	− 0.3356836	− 0.3356823	− 0.3356825	− 0.3356829	− 0.3356828
		Δ	0.05	0.03	− 0.01	0.00	0.00	0.00	0.00	0
Ownhouse	1	β5	0.0074595	0.0073911	0.0067509	0.0062689	0.0062921	0.0062773	0.0062705	0.0062709
		Δ	18.95	17.86	7.65	− 0.03	0.34	0.10	− 0.01	0
Perexilaw	1	β6	− 0.585237	− 0.5852801	− 0.5851538	− 0.5849595	− 0.5849801	− 0.5849752	− 0.5849734	− 0.5849738
		Δ	0.04	0.05	0.03	0.00	0.00	0.00	0.00	0
Literacy	1	β7	− 0.2780723	− 0.277927	− 0.2779039	− 0.278158	− 0.2781428	− 0.2781471	− 0.2781508	− 0.2781504
		Δ	− 0.03	− 0.08	− 0.09	0.00	0.00	0.00	0.00	0
Wage	1	β8	− 0.3008851	− 0.3009183	− 0.3011091	− 0.3012277	− 0.3012232	− 0.3012268	− 0.3012289	− 0.3012288
		Δ	− 0.11	− 0.10	− 0.04	0.00	0.00	0.00	0.00	0
	2	β9	− 0.4023528	− 0.4023962	− 0.4025204	− 0.4025576	− 0.4025593	− 0.4025606	− 0.4025619	− 0.4025618
		Δ	− 0.05	− 0.04	− 0.01	0.00	0.00	0.00	0.00	0
Empowered	1	β10	0.0687065	0.0687084	0.06878	0.0688409	0.0688356	0.0688383	0.068839	0.0688389
		Δ	− 0.19	− 0.19	− 0.09	0.00	0.00	0.00	0.00	
	2	β11	− 0.4141394	− 0.414175	− 0.4141225	− 0.4140116	− 0.4140255	− 0.4140227	− 0.4140218	− 0.414022
		Δ	0.03	0.04	0.02	0.00	0.00	0.00	0.00	0
Cohabdur	1	β14	− 0.2231365	− 0.2231093	− 0.2231121	− 0.2231632	− 0.2231591	− 0.2231613	− 0.2231619	− 0.2231618
		Δ	− 0.01	− 0.02	− 0.02	0.00	0.00	0.00	0.00	0
	2	β15	0.0359486	0.0359922	0.0362362	0.0363868	0.0363811	0.0363856	0.0363878	0.0363877
		Δ	− 1.21	− 1.09	− 0.42	0.00	− 0.02	− 0.01	0.00	0
Residence	1	β16	0.3351694	0.3345385	0.3348971	0.3364416	0.3363268	0.3363573	0.3363743	0.3363723
		Δ	− 0.36	− 0.55	− 0.44	0.02	− 0.01	0.00	0.00	0
State region	1	β18	0.8587015	0.8579745	0.8583721	0.8601331	0.8600154	0.8600562	0.8600763	0.8600745
		Δ	− 0.16	− 0.24	− 0.20	0.01	− 0.01	0.00	0.00	0
	2	β19	1.300386	1.299424	1.29947	1.301329	1.3012	1.301244	1.301263	1.301262
		Δ	− 0.07	− 0.14	− 0.14	0.01	0.00	0.00	0.00	0
	3	β20	1.233859	1.232813	1.2336	1.236344	1.236157	1.236217	1.236249	1.236246
		Δ	− 0.19	− 0.28	− 0.21	0.01	− 0.01	0.00	0.00	0
	4	β21	1.566518	1.565324	1.565393	1.567739	1.567572	1.567617	1.567644	1.567642
		Δ	− 0.07	− 0.15	− 0.14	0.01	0.00	0.00	0.00	0
	5	β22	1.132173	1.131321	1.131443	1.133171	1.133052	1.133093	1.133111	1.13311
		Δ	− 0.08	− 0.16	− 0.15	0.01	− 0.01	0.00	0.00	0
	6	β23	1.921282	1.919887	1.920352	1.923534	1.923363	1.923422	1.923461	1.923458
		Δ	− 0.11	− 0.19	− 0.16	0.00	0.00	0.00	0.00	
	7	β24	0.7046811	0.7039556	0.7047959	0.7069488	0.7068052	0.7068655	0.706888	0.7068863
		Δ	− 0.31	− 0.41	− 0.30	0.01	− 0.01	0.00	0.00	
	8	β25	1.577282	1.576063	1.576114	1.578494	1.578322	1.578366	1.578395	1.578392
		Δ	− 0.07	− 0.15	− 0.14	0.01	0.00	0.00	0.00	
	9	β26	1.446125	1.44494	1.445813	1.448911	1.448719	1.448792	1.448827	1.448824
		Δ	− 0.19	− 0.27	− 0.21	0.01	− 0.01	0.00	0.00	0
	10	β27	1.298739	1.297733	1.297772	1.299724	1.299585	1.299625	1.299647	1.299645
		Δ	− 0.07	− 0.15	− 0.14	0.01	0.00	0.00	0.00	
House*resid	1	β28	0.3957343	0.3958327	0.3964529	0.3968657	0.3968512	0.3968629	0.3968694	0.3968689
		Δ	− 0.29	− 0.26	− 0.10	0.00	0.00	0.00	0.00	0
	variance	σ^2^_uo_	0.5286196	0.528539	0.5373573	0.5459406	0.5454333	0.5456973	0.5458017	0.545793
	change	Δ	− 3.15	− 3.16	− 1.55	0.03	− 0.07	− 0.02	0.00	0
		Log likelihood	− 4824.4213	− 4824.2808	− 4823.052	− 4822.1411	− 4822.1809	− 4822.1659	− 4822.1603	− 4822.1608
		Δ	0.05	0.04	0.02	0.00	0.00	0.00	0.00	

### Appendix 3

See Table 6.

**Table 6:** Multicollinearity test output of covariates

variables	VIF	$\frac{1}{{{\text{VIF}}}}$
Women education	4.08	0.245359
Partner education	3.00	0.333296
Women age	6.15	0.162503
Women empowerment	6.15	0.162503
Cohabitation duration	8.59	0.116407
Perceived existence of law	1.91	0.523432
Literacy status	2.88	0.347195
State region	4.35	0.230138
Own living house	1.42	0.702731
Residence	1.65	0.605906
Mean VIF	4.01	0.249376

### Appendix 4

See Table 7.

**Table 7:** Hosmer–Lemshow goodness of fit test

Group	Prob	Obs_1	Exp_1	Obs_0	Obs_1	Total	Hosmer–Lemeshow chi2(Sig)
1	0.3740	238	229.4	745	753.6	983	9.10 (0.3337)
2	0.5808	464	478.3	517	502.7	981	
3	0.6895	647	638.4	345	353.6	992	
4	0.7388	693	699.1	284	277.9	977	
5	0.7810	764	744.3	213	232.7	977	
6	0.8110	814	833.9	232	212.1	1046	
7	0.8393	774	758.1	144	159.9	918	
8	0.8665	827	837.4	155	144.6	982	
9	0.8909	865	866.6	122	120.4	987	
10	0.9483	890	890.3	87	86.7	977	

## Abbreviations

AIC: Akaike Information Criterion
CSA: Central Statistical Agency
DHS: Demographic Health Survey
EAs: Enumeration Areas
EDHS: Ethiopian Demographic Health Survey
GDP: Growth Domestic Product
ICC: Intraclass Correlation Coefficient
IPV: Intimate Partner Violence
PSU: Primary Sampling Unit
PCV: Proportion Change Variance
SSA: Sub-Saharan Africa
USD: United State Dollar
WHO: World Health Organization

## Declarations

### Availability of data and materials

Data are available from the 2011 Ethiopian Demographic Health Survey Institutional Data Access/ Ethics Committee for researchers who meet the criteria for access to confidential data. Now it is available and can be obtained from the corresponding author.
